# Assembly and engineering of BioBricks to develop an efficient NADH regeneration system

**DOI:** 10.1128/aem.01041-24

**Published:** 2024-12-11

**Authors:** Feng Cheng, Cheng-Jiao Wang, Xiao-Xiao Gong, Ke-Xiang Sun, Xi-Hang Liang, Ya-Ping Xue, Yu-Guo Zheng

**Affiliations:** 1 Key Laboratory of Bioorganic Synthesis of Zhejiang Province, College of Biotechnology and Bioengineering, Zhejiang University of Technology 630365 , Hangzhou, China; 2 The National and Local Joint Engineering Research Center for Biomanufacturing of Chiral Chemicals, Zhejiang University of Technology 12624 , Hangzhou, China; University of Milano-Bicocca, Milan, Italy

**Keywords:** protein engineering, RBS optimization, alcohol dehydrogenase, NADH regeneration system, biocatalytic system

## Abstract

The cofactor regeneration system plays a crucial role in redox biocatalysis for organic synthesis and the pharmaceutical industry. The alcohol dehydrogenase (ADH)-based regeneration system offers a promising solution for the *in situ* regeneration of NAD(P)H. However, its widespread use is hindered by low activity and poor expression of ADH in *Escherichia coli*. Herein, the BioBricks (promoter, ribosome binding site [RBS], functional gene, and terminator) were assembled and engineered to constitute an efficient NADH regeneration system. The semi-rational design was employed to enhance the catalytic efficiency of *Gst*ADH (an ADH from *Geobacillus stearothermophilus*), resulting in a beneficial *Gst*ADH variant with a 2.1-fold increase in catalytic efficiency. Furthermore, the RBS optimization was used to increase the expression of ADH genes, leading to the identification of an RBS with a 3.2-fold increased translation rate. Using this developed system, the NADH generating velocity reached more than 2 s^−1^ even toward 0.1 mM NAD^+^, indicating that it is the most promising NADH regeneration so far. Finally, the engineered system was utilized for the asymmetric biosynthesis of l-phosphinothricin (a chiral herbicide), with a high yield (>95%).

**IMPORTANCE:**

The alcohol dehydrogenase (ADH)-based coenzyme regeneration system serves as a useful tool in redox biocatalysis. This system effectively replenishes NAD(P)H by utilizing isopropanol as a substrate, with the added advantage of easily separable acetone as a by-product. Previous studies focused on discovering new *adh* genes and engineering the ADH protein for higher catalytic efficiency, neglecting the optimization of other gene components. In this study, a remarkably efficient NADH regeneration system was developed using BioBricks assembly for system initialization. The ADH engineering was used to enhance catalytic efficiency, and RBS optimization for elevated ADH expression, which resulted in not only a 2.1-fold increase in catalytic efficiency but also a 3.2-fold increase in translation rate. Together, these improvements resulted in an overall 6.7-fold enhancement in performance. This system finds application in a wide range of NADH-dependent biocatalysis processes and is particularly advantageous for the biosynthesis of fine chemicals.

## INTRODUCTION

Redox reactions play a pivotal role in organic synthesis, yet their application in large-scale enzymatic synthesis of high-value chemicals is frequently constrained by the costliness of cofactors (NADH or NADPH) (1). Given that NADH is more cost-effective and stable than NADPH in industrial settings, the preference leans toward NADH-driven biocatalytic systems for industrial applications. Therefore, there is a pressing need to establish a highly efficient system for *in situ* regeneration of NADH to facilitate environmentally friendly chemical synthesis.

The reduction of NAD^+^ to NADH for this purpose has primarily been achieved through using enzymatic processes (2, 3), chemical reactions (4, 5), photochemical methods (6–8), or electrochemical approaches (9–11). Nevertheless, numerous chemical pathways face challenges due to complicated reaction conditions, limited turnover numbers, the use of costly and/or hazardous reagents, and the occurrence of undesirable side products. Consequently, these methods are not favored for commercial or preparative purposes. On the other hand, biochemical approaches employing enzymes as catalysts have proven to be more effective and versatile (12), including the use of glucose dehydrogenase (GDH) (13), alcohol dehydrogenase (ADH) (14), phosphite dehydrogenase (PDH) (15), formate dehydrogenase (FDH) (16), and hydrogenase (17). GDH, boasting a notable advantage with its high specific activity (200–500 U/mg at 25°C–30°C) (18), encounters challenges associated with substantial co-substrate consumption and difficulties in by-product separation. In contrast, PDH and FDH utilize cost-effective co-substrates for NADH regeneration, avoiding by-product issues, albeit with lower specific activity (FDH, 5–20 U/mg at 25°C–30°C; PDH, 5 U/mg at 25°C) (19). Furthermore, the applications of hydrogenases remain in their early stages due to their low stability and activity (17). Nevertheless, ADH with a comparable specific activity (0.18–10.3 U/mg at 25°C–65°C) (20–22) and easy by-product separation process when using isopropanol as cheap H-donor is of considerable commercial value as a catalyst for NADH regeneration in the synthesis and/or biotransformation of valuable compounds. However, despite the abundance of literature focusing on the utilization of ADHs in the synthesis of chiral alcohols, there has been limited attention given to their role in recycling NADH coenzyme. Additionally, recent progress in enzyme engineering technologies (23, 24) has successfully enhanced certain aspects of ADH performance, such as substrate specificity, enantioselectivity, and catalytic activity in the synthesis of chiral alcohols. These developments inspire us to create an efficient ADH-driven NADH regeneration system.

BioBricks are DNA sequences that conform to a restriction-enzyme assembly standard (25). These building blocks are used to design and assemble larger synthetic biological constructs, which can be incorporated into living cells, such as *Escherichia coli*, to construct new biological systems (26). The BioBricks parts of the ADH-driven NADH regeneration system include the promoter, ribosome binding site (RBS), functional gene, and terminator. Previous studies have primarily focused on discovering new *adh* genes and engineering the ADH protein for higher catalytic efficiency, often neglecting the optimization of other gene components such as promoters, ribosome binding sites (RBS), and terminators. In this study, biological components were assembled to constitute an efficient NADH regeneration system and were engineered not only on the functional ADH gene but also on the RBS part. Through two rounds of semi-rational design, a beneficial variant, *Gst*ADH^E107S+S284T^, was obtained, resulting in a 2.1-fold increase in catalytic efficiency. Subsequently, different RBS sequences were redesigned, matching the *adh* gene, to enhance the expression of *adh* gene in *E. coli*. Moreover, the NADH regeneration system was employed in the asymmetric biosynthesis of L-phosphinothricin (L-PPT), a chiral amino acid and herbicide.

## RESULTS AND DISCUSSION

### Assembling BioBricks for the constitution of an NADH regeneration system

**Fig 1:**
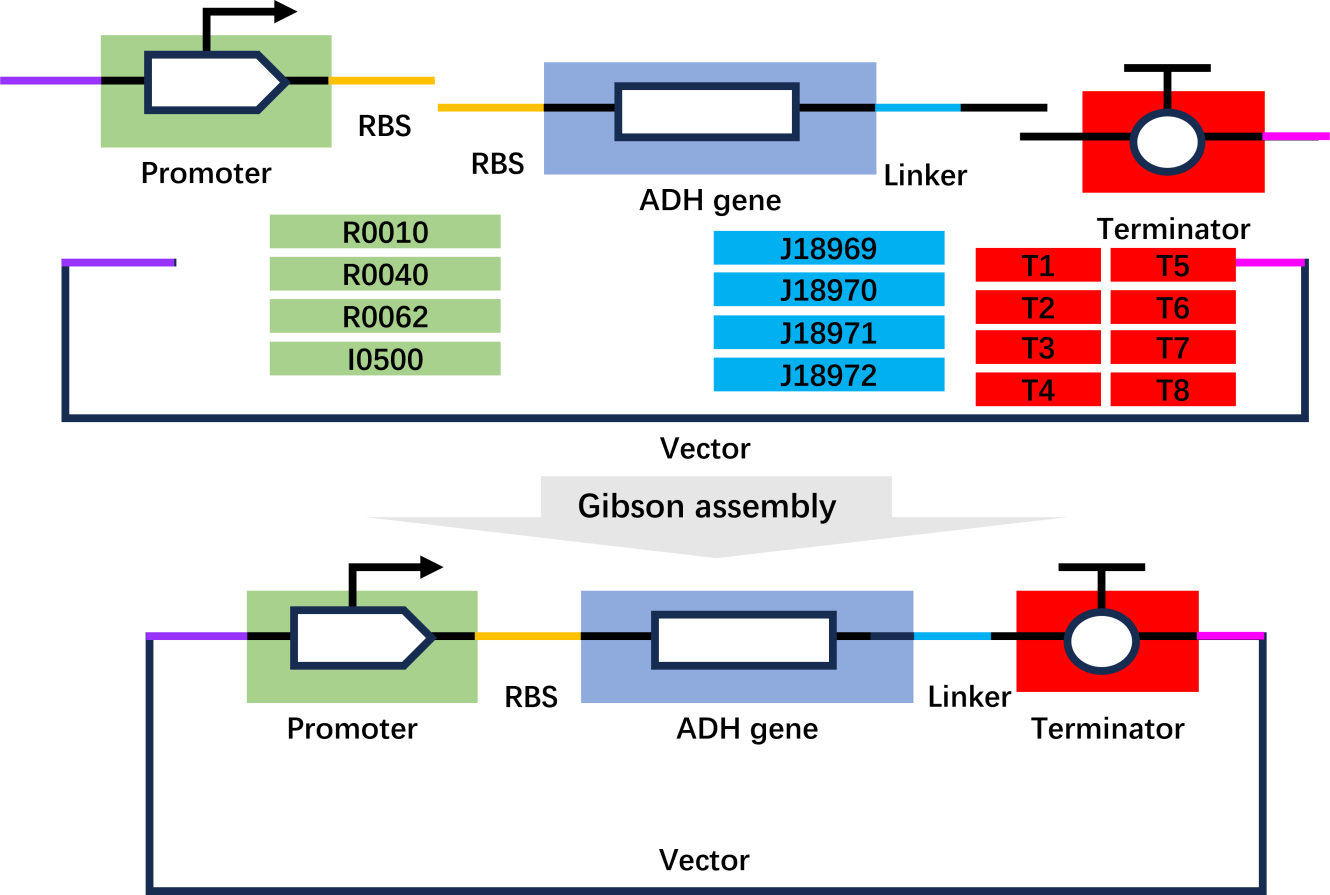
The Gibson assembly-based three-gene randomization methodology. Independent assembly of randomized circuit is randomized with same RBS and ADH gene and with different promoters (R0010, R0040, R0062, and I0500), different linkers (J18969, J18970, J18971, and J18972), and different terminators (J61048, B0024, J18961, J18962, B0015, B0014, J18963, and B0025).

Initially, the *Gst*ADH gene was codon-optimized and integrated into the pETduet vector. However, the resulting construct (pETduet-*Gst*ADH) did not display satisfactory activity (lower than <2 U/mg), and SDS-PAGE analysis revealed low expression of *Gst*ADH. This prompted us to first focus on genetic improvements, including expression and RBS optimization. A randomized assembly approach was employed using BioBricks, involving four promoters, four linkers, and eight transcriptional terminators to construct randomized one-gene circuits via Gibson assembly (Fig. 1). The individual components, including promoters, coding sequences (after codon optimization), and transcriptional terminators, were PCR-amplified with a standardized overlap, facilitating their seamless assembly during the reaction (Table S1). The promoter included a left overlap with a vector sequence and a right overlap with an RBS. Similarly, the coding sequence (NAD^+^-dependent ADH gene from *Geobacillus stearothermophilus*, *Gst*ADH, WP_044744228.1) exhibited a left overlap with the complementary RBS sequence and a right overlap with a 22-base pair “linker” sequence. The terminator displayed a left overlap with the complementary “linker” sequence and a right overlap with the vector suffix. Finally, the vector integrated the complementary prefix and suffix sequences as the left and right overlaps, respectively. To introduce variability in the assembly process, instead of incorporating only one type of component per assembly reaction, multiple fragments of the same component type were combined in a single assembly reaction. Following assembly, the resulting DNA constructs were introduced into *E. coli* BL21 competent cells through transformation. Subsequently, the transformed cells were plated, and the colonies obtained were subjected to activity tests.

Theoretically, a total of 128 potential combinations of different promoters, linkers, and terminator sequences (4 × 4 × 8) were generated. Experimentally, 1,000 colonies were selected from the agar plates, which covers >98% of the generated library. The volume activity of ADH was used as the index for the evaluation of the assembled gene constructions. After the first-round screening, the top 5% of colonies (198 colonies) with activity greater than 18 U/mL were rescreened. Ultimately, one gene construct, pLacZYA-RBSP-*Gst*ADH^WT^-T6, exhibiting high activity (≈30 U/mL ADH), was selected. Since no mutations occurred in the *ADH* (functional gene) during BioBrick assembly, and all changes were in the promoter and terminator regions, the increase in ADH activity is attributed to enhanced protein expression. The expression yield increased from approximately 5% to 25% of the total soluble proteins.

### Semi-rational design of ADH and the activity enhancement

**Fig 2:**
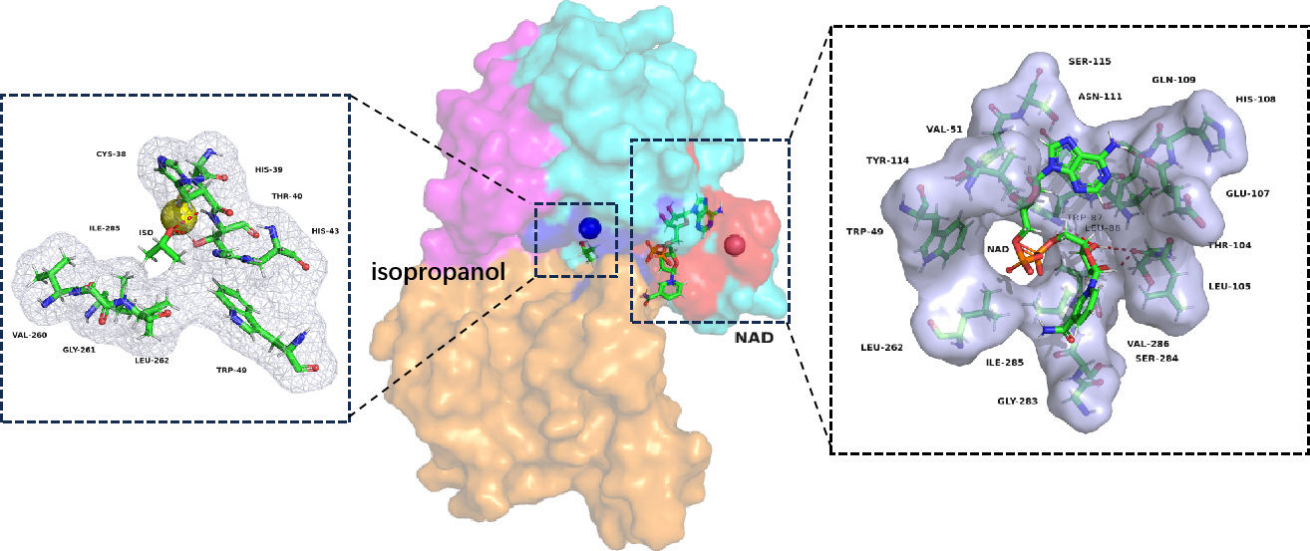
The docked conformation of ADH with NAD^+^ and isopropanol. The comprehensive model of ADH with NAD and isopropanol (iso) is depicted in the center, highlighting the isopropanol-binding pocket in blue and the NAD-binding pocket in red. The dashed box on the left specifically illustrates the isopropanol-binding pocket, while the dashed box on the right focuses on the NAD-binding pocket. Hydrogen bonds are indicated by red dashed lines. The cofactor-binding domain, consisting of residues 150–285, is presented in orange, while residues 1–149 and 286–339 form the catalytic domain, with the former displayed in sky blue and the latter in purplish red.

The semi-rational design is a classical approach in protein engineering. Here, we used this approach to engineer the functional *adh* gene part. The *Gst*ADH enzyme exhibits a tetrameric structure (PDB ID 1RJW), with each subunit comprising two distinct domains: residues 150–285 form the cofactor-binding domain (colored orange), while residues 1–149 (sky blue) and 286–339 (purple-red) constitute the catalytic domain. A deep cleft separates these two domains, providing a site for the catalytic zinc atom. The NAD and isopropanol molecules were extracted from the crystal structure of a bacterial zinc-dependent alcohol dehydrogenase (PDB 3s2e) and subsequently docked into the *Gst*ADH receptor. Cofactor-binding domains tightly associate with adjacent subunits, forming an extended plane along the tetramer’s sides. Isopropanol is situated within a cavity surrounded by the amino acid residues Cys38, His39, Thr40, His43, Trp49, Val260, Gly261, Leu262, and Ile285. The oxygen atom of isopropanol forms two hydrogen bonds with Thr40. NAD is enveloped by a pocket formed by amino acid residues Trp49, Tyr114, Val51, Ser115, Asn111, Gln109, His108, Glu107, Thr104, Leu105, Val286, Ser284, Gly283, Ile285, Leu262, Trp87, and Leu88 (Fig. 2). NAD forms two, two, and one hydrogen bonds with Trp87, Thr104, and Glu107, respectively. Therefore, these 17 positions were selected as potential beneficial mutation sites (using NDT as codon). In total, 1,700 colonies (100 for each position) were screened, and the activities of the purified enzymes were compared. Twenty-three variants exhibited more than 1.5-fold higher activity than *Gst*ADH^WT^, with two beneficial variants (*Gst*ADH^E107S^ and *Gst*ADH^S284T^) showing over twofold higher activity (*Gst*ADH^E107S^: 51.2 U/mg; *Gst*ADH^S284T^: 44.9 U/mg; and *Gst*ADH^WT^: 29.6 U/mg). Furthermore, the combination variant *Gst*ADH^E107S+S284T^ showed the highest activity (59.8 U/mg).

Prior to mutation, NAD molecules interacted with E107, W87, and T104 of *Gst*ADH^WT^ through hydrogen bonding. After mutation, the two hydrogen bonds formed with W87 were reduced to one, while a new hydrogen bond with N111 emerged. The total hydrogen bond length decreased, strengthening the interactions between the receptor and ligand (Fig. S1). This may potentially enhance the stability of the enzyme. Furthermore, the mutation led to a larger opening at the interface pocket, facilitating ligand entry and exit from the active pocket. At the V286 position, Val mutated to Phe, introducing a highly stable benzene ring due to the shift from an aliphatic to an aromatic amino acid. This change could potentially enhance the stability of the enzyme protein. The mutation at the S284 position, where Ser was mutated to Thr, involved a shift from a highly polar amino acid to a less polar one (Fig. S1). The change in polarity might be a contributing factor to the improvement in enzyme activity.

### RBS redesigned and ADH expression level test

The Gibbs free energy difference (*∆G*_t0t_) and the translation rate were calculated by RBS Calculator. The calculation of *∆G*_t0t_ incorporates several factors, including the energy released during the hybridization of the 16S rRNA binding site with the mRNA subsequence, the energy released when the start codon hybridizes with the anticodon loop of the initiating tRNA, and the free energy loss resulting from the non-optimal physical distance between the 16S rRNA binding site and the original codon. It also considers the energy required to unfold the mRNA sequence into its most stable secondary structure, a process that can be analyzed and computed using the RNAfold WebServer. Approximately 10^6^ RBS sequences were tested *in silico*, resulting in seven RBS variants with predicted higher translation rates (Table S2). The corresponding energies (*∆G*_total_, *∆G*_mRNA:rRNA_, *∆G*_spacing_, *∆G*_standby_, *∆G*_start_, and *∆G*_mRNA_) and translation rates are detailed in Table S3, with *∆G*_total_ ranging from −9.14 to −13.19 kcal/mol. These RBS sequences were replaced by the origin one in the assembled construct (pLacZYA-RBS^P^-*Gst*ADH^WT^-T6). The ADH gene expression was detected (Fig. S2), and the RBS AR2 led to the highest expression level of ADH. The AR2 is a 35-bp-long RBS sequence with a characteristic Shine-Dalgarno sequence of “AGGAGGT” and a 3′-tail sequence of “ATTATT.” The predicted translation rate of AR2 was 3.2-fold higher than that of the original RBS in the assembled construct (from 152,485.59 to 487,953.90, Table S3). Consequently, the optimal RBS was chosen to enhance the expression of ADH in *E. coli* cells.

### Characterization of the ADH-based NADH regeneration system

The effects of different pHs (5.0–10.0) of Britton–Robinson buffer solutions on *Gst*ADH-based NADH regeneration systems (pLacZYA-RBS^P^-*Gst*ADH-T6) were examined. The optimal reaction pH of the developed NADH regeneration system is around 8.5 (Fig. S3), and the system preferably favors reactions in alkaline environments. The relative activity of *Gst*ADH^E107S/S284T^ remained >60% across the pH range from 7.5 to 10.0. However, in weakly acidic environments (pH 5.0–7.0), *Gst*ADH activity dropped to less than 40% of its activity at pH 8.5.

The thermal stability of enzymes is another indispensable characteristic in industrial production. Temperature effects on the ADH-based NADH regeneration system were investigated by assessing the thermal stability of ADH at three temperatures (35°C, 55°C, and 65°C) (Fig. S4). The half-lives of *Gst*ADH^WT^ at 35°C, 55°C, and 65°C are 15.8, 3.8, and 1.3 hours, respectively. For *Gst*ADH^E107S^, the half-lives at these temperatures are 11.5, 5.4, and 1.2 hours, respectively. At 55°C, the half-life increased to 142% compared to the *Gst*ADH^WT^. The half-lives of *Gst*ADH^E107S/S284T^ at 35°C, 55°C, and 65°C are 10.4, 3.0, and 1.6 hours, respectively, with an increased half-life at 65°C compared to the wild type. *Gst*ADH^E107S/S284T^ and *Gst*ADH^E107S^ show different half-life advantages at various temperatures. Both the 107 and 284 sites are near the NAD binding pocket. Although the mutation at site 107 strengthens the interaction between the enzyme and NAD, the mutation from serine (S) to threonine (T) at site 284 results in a change from a highly polar to a less polar residue. This polarity change may account for the different half-life advantages of the combined mutations.

To explore the applicability of this NADH regeneration system, the kinetic parameters for alcohol dehydrogenase *Gst*ADH^WT^ and its variants were determined concerning isopropanol (2–400 mM) and NAD^+^ (0.1–10 mM) (Table 1; Fig. S4). Compared to the wild-type *Gst*ADH, *Gst*ADH^E107S^ exhibited significantly increased affinity for NAD^+^ (*K*_*m*_ value decreased from 0.76 to 0.36 mM), enabling efficient conversion to NADH at lower NAD^+^ concentrations. Its catalytic efficiency, *k*_cat_/*K*_*m*_, was 17.75 mM^−1^s^−1^, approximately 1.7 times higher than that of the wild type (10.86 mM^−1^s^−1^). Importantly, it demonstrated excellent conversion efficiency at NAD concentrations as low as 0.1 mM (*v* = 1.8 s^−1^), indicating a broader range of applicability for this NADH regeneration system. *Gst*ADH^E107S^ also exhibited significantly increased affinity for isopropanol, with its *K*_*m*_ value decreasing from 14.86 mM (*Gst*ADH^WT^) to 6.04 mM. Upon introducing the S284T mutation to *Gst*ADH^E107S^, *Gst*ADH^E107S+S284T^ showed similar NAD^+^ affinity to *Gst*ADH^E107S^ and better isopropanol affinity comparable to *Gst*ADH^WT^. It achieved the highest catalytic efficiency, measured at 22.81 (mM^−1^s^−1^). The lower limit of the applicable range for the NADH regeneration system (pLacZYA-RBS^P^-*Gst*ADH^E107S+S284T^-T6) based on *Gst*ADH^E107S+S284T^ was reduced to 0.1 mM (*v* = 2.0 s^−1^).

**TABLE 1:** Kinetic parameters of *Gst*ADH and its variants

Variant	Substrate	*K*_*m*_ (mM)	*k*_cat_ (s^−1^)	*k*_cat_/*K*_*m*_ (mM^−1^s^−1^)
*Gst*ADH^WT^	NAD^+^	0.76 ± 0.01	8.28 ± 0.01	10.86 ± 0.02
	Isopropanol	14.86 ± 1.51	8.32 ± 0.04	0.56 ± 0.01
*Gst*ADH^S284T^ (M1)	NAD^+^	0.69 ± 0.27	9.24 ± 0.24	13.39 ± 1.02
	Isopropanol	9.37 ± 1.20	8.24 ± 0.32	0.88 ± 0.01
*Gst*ADH^E107S^ (M2)	NAD^+^	0.36 ± 0.23	6.35 ± 0.12	17.75 ± 0.13
	Isopropanol	6.04 ± 9.26	6.34 ± 0.17	1.05 ± 0.02
*Gst*ADH^E107S+S284T^ (M3)	NAD^+^	0.39 ± 0.31	8.89 ± 0.24	22.81 ± 0.30
	Isopropanol	7.19 ± 1.46	8.08 ± 0.55	1.12 ± 0.01

The kinetic data for the reported ADH are summarized in Table 2. In comparison, the *Gst*ADH^E107S+S284T^ variant demonstrated a lower *K*_*m*_ value (0.39 mM) and a *k*_cat_ value (8.89 s^−1^), indicating a catalytic efficiency surpassing that of most ADHs (Table 2). Furthermore, the kinetic data of three commonly used cofactor recycling system enzymes (ADH, GDH, and FDH) toward NAD^+^ were compared. Overall, ADH and FDH showed lower catalytic efficiency than GDH (22.81 vs 702 s^−1^ mM^−1^) (Table S4). However, ADH- and FDH-mediated cofactor recycling systems produce clean products without the formation of gluconic acid, making them widely used. Furthermore, ADH’s catalytic efficiency is slightly higher than that of FDH (Table S4). Importantly, the *Gst*ADH^E107S+S284T^ demonstrated high substrate affinity toward NAD+, ensuring high activity of ADH at low substrate concentrations, thus providing an excellent enzyme component for the cofactor regeneration system.

**TABLE 2:** Kinetic parameters of native NAD^+^-dependent ADHs and their variants

Enzyme	NAD^+^				Isopropanol		Reference
	*K*_*m*_ (mM)	*k*_cat_ (s^–1^)	*k*_cat_/*K*_*m*_ (mM^−1^s^−1^)	*K*_*m*_ (mM)	*k*_cat_ (s^–1^)	*k*_cat_/*K*_*m*_ (mM^−1^s^−1^)	
*Aeropyrum pernix* ADH^WT^	0.001	0.4	380	2.44	0.24	0.097	(27)
*Mycobacterium* sp. ADH^WT^	0.02	1	80	ND^*a*^	ND	ND	(28)
*Thermococcus kodakarensis* ADH^WT^	0.127	30.8	243	ND	ND	ND	(29)
*Sulfolobus acidocaldarius* ADH^WT^	0.44	3.7	8.4	ND	ND	ND	(30)
*Thermus thermophilus* ADH^WT^	0.24	0.84	3.5	ND	ND	ND	(31)
*Leifsonia* sp. ADH^WT^	0.12	ND	ND	57.5	ND	ND	(23)
*Euglena gracilis* ADH^WT^	0.39	ND	ND	20.3	ND	ND	(32)
*Scaptodrosophila lebanonensis* ADH^WT^	0.028	ND	ND	1.1	ND	ND	(33)
*Homo sapiens* ADH^WT^	ND	ND	ND	560	0.75	0.001	(34)
Equus caballus ADH^WT^	ND	ND	ND	9	0.58	0.064	(35)
*Methylorubrum extorquens* ADH^WT^	ND	ND	ND	3.42	ND	ND	(21)
Fragaria × ananassa ADH^WT^	ND	ND	ND	11.46	ND	ND	(21)
*Corynebacterium glutamicum* ADH^WT^	ND	ND	ND	19.6	ND	ND	(36)
*Saccharolobus solfataricus* ADH^WT^	0.2	0.28	1.4	0.6	0.25	0.417	(21)
*Saccharolobus solfataricus* ADH^N249Y^	12.4	50	4.032	ND	ND	ND	(37)
*Saccharolobus solfataricus* ADH^W95L^	4	0.52	0.13	ND	ND	ND	(38)
*Saccharolobus solfataricus* ADH^W95L/N249Y^	50	21	0.42	ND	ND	ND	(38)
*Pyrococcus furiosus* ADH^WT^	0.6	2.6	4.333	ND	ND	ND	(39)
*Pyrococcus furiosus* ADH^G211C^	0.48	1	2.083	ND	ND	ND	(40)
*Pyrococcus furiosus* ADH^G211S^	0.1	4	40	ND	ND	ND	(40)
*Pyrococcus furiosus* ADH^K249G/H255R^	0.46	15	32.609	ND	ND	ND	(41)
*Geobacillus stearothermophilus* ADH^WT^	0.45	33	73	57.8	287	5	(42)
*Geobacillus stearothermophilus* ADH^W87A^	0.2	3.2	16	ND	ND	ND	(43)
*Geobacillus stearothermophilus* ADH^Y25A^	1	5.1	5.1	ND	ND	ND	(44)
*Geobacillus stearothermophilus* ADH^V260A^	10	1.9	0.19	ND	ND	ND	(44)
*Geobacillus stearothermophilus* ADH^Y25A/W49F/W87F/V260A^	14.8	2.5	0.169	ND	ND	ND	(44)
*Geobacillus stearothermophilus* ADH^Y25A/W49F/W167Y/V260A^	9.7	1.9	0.196	ND	ND	ND	(44)

^*a*^ ND, not detected.

### Case application: biosynthesis of L-PPT using the developed cofactor regeneration system

**Fig 3:**
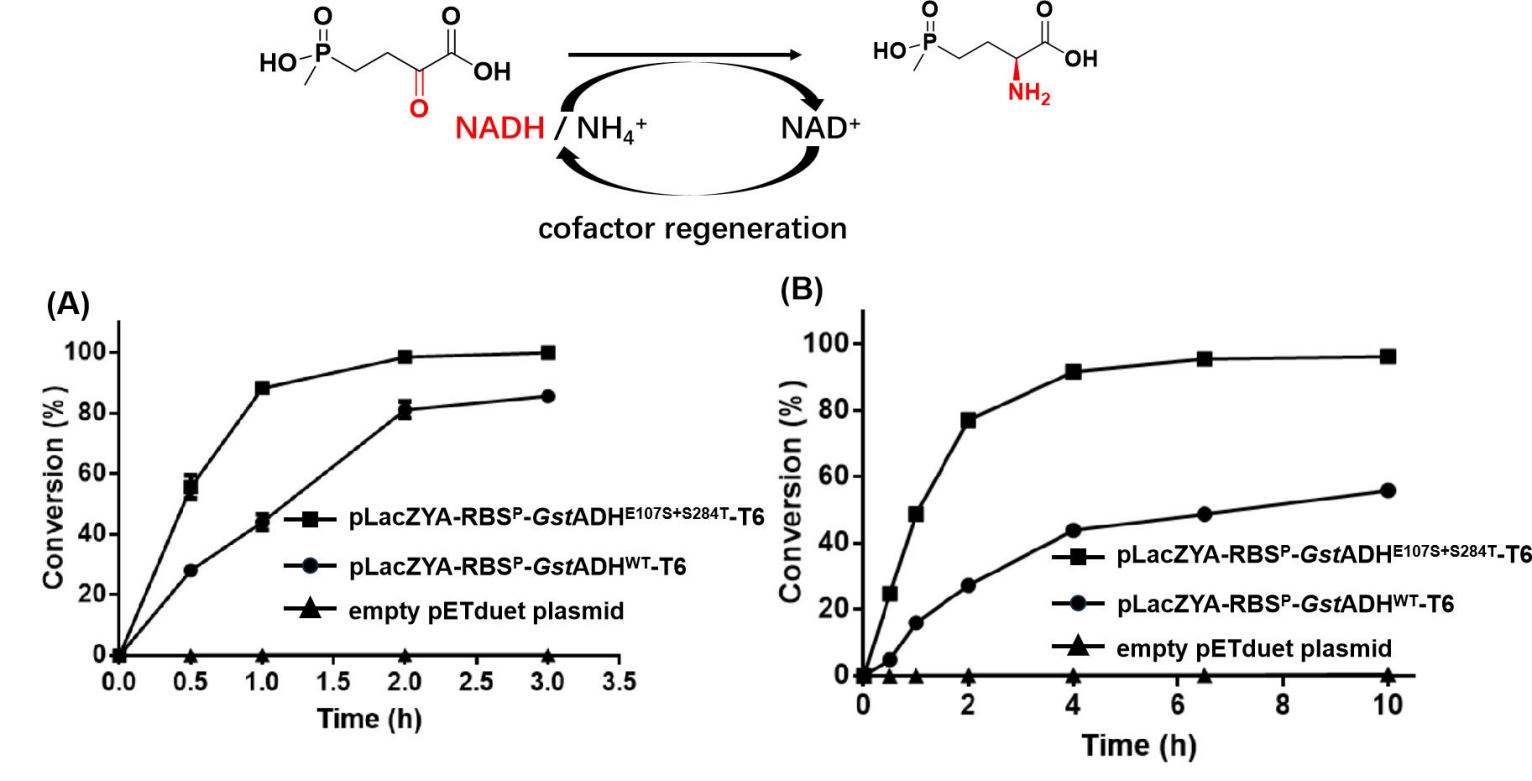
The principle of the ADH-base cofactor regeneration system and its case application. In the experiment group, the blank control group consists of *E. coli* harboring an empty vector (pETduet) as the biocatalyst. (**A**) The time course of biotransformation of 100 mM PPO by employing original gene construction-driven NADH regeneration system (pLacZYA-RBS^P^-*Gst*ADH^WT^-T6) and engineered gene construction-driven NADH regeneration system (pLacZYA-RBS^P^-*Gst*ADH^E107S+S284T^-T6). (**B**) The time course of biotransformation of 300 mM PPO by employing original gene construction-driven NADH regeneration system (pLacZYA-RBS^P^-*Gst*ADH^WT^-T6) and engineered gene construction-driven NADH regeneration system (pLacZYA-RBS^P^-*Gst*ADH^E107S+S284T^-T6).

To assess the practical utility of the developed cofactor regeneration system, we linked the pLacZYA-RBS^P^-*Gst*ADH^WT^-T6 with an engineered glutamate dehydrogenase (GluDH^A164G^, a previously identified enzyme for L-PPT synthesis [16]) (Fig. 3). In the presence of 100 mM (2-oxo-4-{(hydroxy)(methyl)phosphinyl butyric acid}) PPO as a substrate, with the addition of 250 mM isopropanol and 0.06 mM NAD^+^ in the reaction system under pH 8.5 and 35°C, the mutant group achieves a conversion of 90% after 1 hour of catalysis. After 2 hours, the catalytic reaction is complete, reaching 100% conversion, while the original gene construction-driven NADH regeneration system (pLacZYA-RBS^P^-*Gst*ADH^WT^-T6) only achieves a 40% conversion (Fig. 3A). Under the reaction conditions of 300 mM PPO, 600 mM isopropanol, 0.3 mM NAD^+^, 15 g/L bacteria, and pH 8.5, the mutant group achieves a 90% conversion after 4 hours of catalysis, whereas the control group only achieves a 40% conversion (Fig. 3B). After 6 hours of reaction and later purification, a yield of 95% l-PPT (100% conversion) is reached, while the control group only achieves a 49% conversion. Meanwhile, using *E. coli* harboring an empty vector (pET) as the biocatalyst (blank control group), no conversion was observed (Fig. 3). The reaction mixture was collected and subjected to centrifugation for conversion, followed by direct spray drying. Significantly, the purity of the obtained L-PPT exceeded 90% and high enantioselectivity (>99% *ee*) (NMR spectra of L-PPT are shown in Fig. S6 and S7). Therefore, the developed ADH-based cofactor regeneration system, particularly for NADH, demonstrates significant potential for the large-scale synthesis of fine chemicals in industrial applications.

## MATERIALS AND METHODS

### Experimental materials

The D,L-phosphinothricin (95%) was purchased from Bidepharm (Shanghai, China). NADH and NAD^+^ were obtained from J&K Scientific (Shanghai, China). Taq DNA polymerase, Phanta max super-fidelity DNA polymerase, and a one-step cloning kit were procured from Vazyme (Nanjing, China). The PCR clean-up kit and plasmid miniprep kit were acquired from Axygen (Shanghai, China). DpnI was purchased from Thermo Scientific (Shanghai, China). *E. coli* BL21(DE3) competent cells were obtained from TransGen Biotech (Beijing, China) for gene expression and protein production. All other chemicals and reagents were sourced commercially.

### Construction of mutant libraries

Mutations were incorporated into the ADH derived from *Geobacillus stearothermophilus* (*Gst*ADH, Sequence ID: WP_044744228.1) through site-directed mutagenesis or site saturation, employing a QuikChange Mutagenesis Kit along with Phanta Max Super-Fidelity DNA Polymerase (Vazyme, Nanjing, China). The primers utilized are detailed in Tables S5 and S6. The PCR conditions for the fragment were as follows: 95°C for 5 min (95°C for 30 s, 60°C–70°C for 30 s, and 72°C for 4 min), repeated for 30 cycles, and a final extension at 72°C for 5 min (23). After electrophoresis, the PCR products were examined on an agarose gel and subsequently treated with 1 µL of DpnI for 2 hours at 37°C to eliminate the template plasmids. Following this, *E. coli* BL21(DE3) competent cells were transformed with the PCR products using a chemical method.

### Redesign of ribosome binding site sequences

Ribosome binding site sequences before the alcohol dehydrogenase gene were redesigned using the RBS Calculator (45–48). In the redesign procedure, the translation initiation rate and the host for ADH gene expression were taken into consideration. Based on the results, seven RBS sequences were selected and used to replace the original RBS sequence from pETDuet vector (for primers, see Table S2).

### Screening of mutant libraries

After obtaining the transformants, single colonies were selected and inoculated into 600 µL of LB medium (with 50 µg/mL kanamycin) in each well of a 96-well plate. The cultures were then incubated for 12 hours at 37°C with agitation at 180 rpm. Following this, 200 µL of seeding culture from each well was transferred to a new 96-well plate containing 600 µL of LB medium (supplemented with 50 µg/mL kanamycin and 0.1 mM IPTG) and cultivated at 28°C for 14 hours (180 rpm). Upon centrifugation at 8,000 rpm for 10 min, the supernatant was discarded, and the 96-well plate containing bacteria was placed at the bottom of each well into a −20°C refrigerator for freezing and storage for more than 4 hours. Subsequently, the cells were re-suspended in 300 µL of 0.1 M phosphate buffer (pH 8.5) and incubated at 35°C for 30 min. Afterward, 100 µL of the resulting supernatant was transferred into fresh plates for assay purposes. To initiate the reaction, 100 µL of the prepared substrate solution, containing 100 mM isopropanol and 1 mM NAD^+^, was added. Upon triggering the reaction, the absorbance at 340 nm was immediately measured using an enzyme meter. The beneficial variants were sequenced and stored in a 30% glycerin solution at −80°C.

### Gibbs free energy calculation for RBS redesign

The RBS Calculator used for the calculation of the Gibbs free energy difference (*ΔG*_t0t_) when the 30S complex assembles on an mRNA transcript sequence computes using the formula:

$\Delta G_{t_{0}t}=\Delta G_{mRNA:rRNA}+\Delta G_{start}+\Delta G_{spacing}-\Delta G_{standby}-\Delta G_{mRNA}$. *∆G*_mRNA_: rRNA is the energy released when the last nine nucleotides of *E. coli* 16S rRNA (3′-AUUCCUCCA-5′) hybridize with the mRNA subsequence at the 16S rRNA binding site (*∆G*_mRNA_ :rRNA < 0). ∆*G*_start_ is the energy released when the start codon hybridizes with the anticodon loop of the initiating tRNA (3′-UAC-5′). ∆*G*_spacing_ accounts for the energy loss due to the non-optimal physical distance between the 16S rRNA binding site and the start codon (*∆G*_spacing_ > 0). The energy for unfolding five amino acids is defined as 0, *∆G*_mRNA_ is the energy required to unfold the mRNA sequence into its most stable secondary structure (*∆G*_mRNA_ < 0), which can be determined using the RNAfold WebServer. *∆G*_standby_ is the energy required to unfold the secondary structure of standby sites (*∆G*_standby_ < 0). The translation speed was calculated by the formula $Rate=k\times e^{\left(-\beta \times \Delta G_{tot}\right)}$.

### Enzyme expression, purification, and validation

Recombinant *E. coli* cells were cultured in 10 mL of LB medium containing 50 µg/mL kanamycin and grown at 37°C with agitation at 180 rpm for 8 hours. Subsequently, 2 mL of the seed solution was transferred into 100 mL of fresh LB medium containing 50 µg/mL kanamycin and incubated for approximately 2 hours, reaching an OD_600_ value of 0.6–0.8. The enzyme was induced by adding IPTG (0.1 mM final concentration) and cultivated at 28°C for 14 hours. The cells were harvested by centrifugation at 8,000 rpm and 4°C for 10 min. For enzyme purification, the collected cells were washed and resuspended in 0.1 M phosphate buffer (pH 8.5), and the enzyme was released through ultrasonic crushing in an ice bath for 20 min. Precipitated impurities were removed by centrifugation for 10 min at 12,000 rpm and 4°C. The supernatant was purified through nickel ion affinity chromatography. The purity and molecular weight of the collected enzyme were analyzed by SDS-PAGE, and the concentration was determined using a BCA protein assay kit (49).

### Enzyme characterizations

For the evaluation of purified *Gst*ADH activity, the reaction mixture consisted of 100 mM isopropanol, 2 mM NADH, and a 1 g/L enzyme solution. Protein concentration was determined using the BCA Protein Assay Kit for subsequent calculation of specific activity and kinetic parameters. One unit of activity (U) was defined as the amount of enzyme required to produce 1 µmol NADH per minute at 35°C and pH 7.5. Specific activity (U/mg) was calculated as the ratio of activity to the amount of enzyme added (mg) (50). The kinetic parameters of *Gst*ADH^WT^ and its variants toward isopropanol and NAD^+^ were measured at 35°C and pH 7.5. The reaction mixture contained 2–400 mM isopropanol and 0.05–10 mM NAD^+^. Using the Michaelis-Menten equation, the turnover number (*k*_cat_) and Michaelis constant (*K*_*m*_) were determined through nonlinear fitting in Origin 2021 .

The optimization of reaction pH was conducted by assessing enzyme activity at 35°C and pH 5–10 (Britton–Robinson buffer). Furthermore, purified enzymes underwent incubation at temperatures of 35°C, 55°C, and 65°C for 24 hours, with samples taken at different time intervals for activity assays. The half-lives (*t*_1/2_) at each temperature were calculated through nonlinear fitting. Temperature stability was analyzed by incubating the purified enzymes at temperatures of 35°C, 55°C, and 65°C for varying durations and determining the residual enzyme activity at different time points. The half-lives (*t*_1/2_) at each temperature were calculated through nonlinear fitting (51).

### Molecular dynamics simulations

The NAD and isopropanol molecules were extracted from the crystal structure of a bacterial zinc-dependent alcohol dehydrogenase (PDB 3s2e) and subsequently docked into the *Gst*ADH receptor. Molecular dynamics (MD) simulations were executed to analyze the complex of *Gst*ADH-NAD-isopropanol and its mutant at 298 K using YASARA software with the Amber11 force field (52). Following the simulations, trajectory analyses were carried out on the MD simulation data utilizing the Visual Molecular Dynamics program to extract root-mean-square deviation, root-mean-square fluctuation, and receptor-ligand binding energy. Trajectory data were collected every 100 ps for subsequent analysis, and the total length of the analysis was 20 ns.

### Construction of a recombinant *E. coli* strain harboring glutamate dehydrogenase and alcohol dehydrogenase genes

We constructed a dual-enzyme co-expression strain combining glutamate dehydrogenase and alcohol dehydrogenase for the asymmetric amination of PPO, aimed at the preparation of L-PPT. The production conditions for the enzymes were systematically optimized. To enhance enzyme production, we conducted optimization of the culture temperature by evaluating catalytic efficiency under various induction temperatures, ranging from 20°C to 30°C. Furthermore, the concentration of the inducer, IPTG, was optimized at 0.025, 0.05, 0.1, 0.2, and 0.3 mM. The incubation time was also optimized by assessing catalytic efficiency after 10, 12, 14, 16, and 18 hours of incubation. The catalytic conditions of the enzyme were further fine-tuned. Reaction pH was optimized by measuring catalytic effects at 35°C and varying pH values from 5 to 10, using Britton–Robinson buffer. The reaction temperature was optimized by measuring the catalytic effect at 35°C, 40°C, 45°C, 50°C, 55°C, and 60°C.

### Asymmetric amination of PPO using the recombinant *E. coli* cells

The asymmetric amination of PPO for the synthesis of L-PPT was performed using lyophilized *E. coli* BL21 cells overexpressing GluDH and ADH. In the GluDH-ADH system, the reaction mixture consisted of 10 g/L cells, 100–300 mM PPO, 100–500 mM isopropanol, and 0.03–1 mM NAD+. Reactions were carried out at a temperature of 55°C and pH 7.5. Samples were collected at various time points from the reaction mixture, and after centrifugation at 12,000 rpm for 1 min, they were analyzed for product concentration and enantioselectivity (24) using high-performance liquid chromatography (HPLC). The isolated products were purified through methanol crystallization, and the crystals underwent XRD and NMR analysis.

### Determination and characterization of substrates and products

The PPO concentration was determined using high-performance liquid chromatography with a C18 column (Unitary C 18, 5 µm, 100 A, 4.6 × 250 mm) at a flow rate of 1.0 mL/min, and detection was carried out at a wavelength of 232 nm. Each 10 µL sample was eluted with a mobile phase comprising 50 mM ammonium dihydrogen phosphate (pH 3.8) buffer containing 0.1% tetrabutyl ammonium bromide and 12% acetonitrile. The column temperature was maintained at 40°C (53). The products containing D-PPT and L-PPT were determined at fluorescence wavelengths of *λ*_ex_ = 340 nm and *λ*_em_ = 450 nm after 5 min of derivatization using *o*-phthalaldehyde and *N*-acetyl-L-cysteine at 30°C in the same HPLC instrument and column. The mobile phase for this analysis consisted of 50 mM ammonium acetate (pH 5.7) and methanol (9:1, vol/vol). The column temperature was maintained at 35°C with a flow rate of 1.0 mL/min. The retention time of D-PPT and L-PPT were 11.3 and 13.9 min, respectively. Furthermore, ^1^H-NMR and ^13^C-NMR analyses were performed for the substrate and product, respectively.

### Crystallization and purification of L-PPT

The reaction solution underwent an initial centrifugation and filtration step to eliminate denatured proteins. Calcium hydroxide (PPO: calcium hydroxide = 1:1, *n*:*n*) was then introduced to the supernatant, which underwent magnetic stirring at 600 rpm and 60°C for 2 hours. Following this, the mixture was centrifuged at 8,000 rpm and 4°C for 10 min to remove sulfate. The resulting supernatant underwent decompression distillation using a rotary evaporator with a vacuum of 0.1 MPa under 60°C for 6 hours. The gel obtained from spin distillation was dissolved by adding an appropriate amount of methanol (initial reaction solution: methanol volume ratio of 1:1.2) and rotated overnight at 80 rpm to enhance dissolution using a spin distillation apparatus. The methanol solution was obtained after filtration. The pH of the methanol solution was adjusted to 4.2 using concentrated sulfuric acid, and the crystallization process was divided into three steps. First, the L-PPT was dried in an oven at 65°C. Second, it was allowed to stand at room temperature for about 4 hours, and after complete delamination of the solution, the crystals were obtained by filtration. Finally, the solution was subjected to magnetic stirring for 4 hours under 600 rpm and 25°C, and the crystals were obtained by filtration. The collected crystals were then dried in an oven to obtain the crystal powder of L-PPT, which was subsequently analyzed by HPLC. An appropriate amount of crystal powder was sent to the Analysis and Testing Center of Zhejiang University of Technology for XRD detection.

## SUPPLEMENTAL MATERIAL

Supplemental material aem.01041-24-s0001.docx Tables S1 to S6; Figures S1 to S7.

ASM does not own the copyrights to Supplemental Material that may be linked to, or accessed through, an article. The authors have granted ASM a non-exclusive, world-wide license to publish the Supplemental Material files. Please contact the corresponding author directly for reuse.
