# Supplementary material for: Assembly and engineering of BioBricks to develop an efficient NADH regeneration system
Source: Appl Environ Microbiol. 2024 Dec 11;91(1):e01041-24. doi: 10.1128/aem.01041-24 (PMC11784351; doi:10.1128/aem.01041-24)
Supplement: Supplemental material — Tables S1 to S6; Figures S1 to S7. [file aem.01041-24-s0001.docx]

**Supplementary material**

**Assembly and Engineering of BioBricks to Develop an Efficient NADH Regeneration System**

Feng Cheng^a,b^, Cheng-Jiao Wang^a,b^, Xiao-Xiao Gong^a,b^, Ke-Xiang Sun, ^a,b^, Xi-Hang Liang^a,b^, Ya-Ping Xue^a,b,^*, Yu-Guo Zheng^a,b^

^a^ Key Laboratory of Bioorganic Synthesis of Zhejiang Province, College of Biotechnology and Bioengineering, Zhejiang University of Technology, Hangzhou 310014, P. R. China

^b^ The National and Local Joint Engineering Research Center for Biomanufacturing of Chiral Chemicals, Zhejiang University of Technology, Hangzhou 310014, P. R. China

* Corresponding author: Prof. Ya-Ping Xue, College of Biotechnology and Bioengineering, Zhejiang University of Technology, 18 Chaowang Road, Hangzhou 310014, China

Tel: +86-571-88320630; Fax: +86-571-88320630

E-mail: xyp@zjut.edu.cn

**Table S1** Parts and vectors used for Randomized BioBrick Assembly

| **Part ID** | **Short ID** | **Part Type** | **Description** |
| --- | --- | --- | --- |
| R0010 | pLacZYA | Promoter | pLacZYA promoter, repressed by Lacl, inducible by IPTG |
| R0040 | pTetR | Promoter | pTetR promoter, repressed by TetR, inducible by aTc |
| R0062 | pLuxR | Promoter | pLuxR promoter, activated by LuxR when AHL is present |
| I0500 | pBAD | Promoter | pBAD promoter, activated by arabinose when AraCis present |
| B0031 | RBS^P^ | RBS | RBS from pETDuet plasmid |
| J18969 | LinkerA | Linker | Coding Sequence-Terminator Linker A |
| J18970 | LinkerB | Linker | Coding Sequence-Terminator Linker B |
| J18971 | LinkerC | Linker | Coding Sequence-Terminator Linker C |
| J18972 | LinkerD | Linker | Coding Sequence-Terminator Linker D |
| J61048 | T1 | Transcriptional Terminator | Derived from the T1 terminator from rnpB gene of *E. coli* MG1655 |
| B0024 | T2 | Transcriptional Terminator | Reverse sequence of B0014 |
| J18961 | T3 | Transcriptional Terminator | bla terminator from DNA2.0 plasmid |
| J18962 | T4 | Transcriptional Terminator | rrnB1 terminator from DNA2.0 plasmid |
| B0015 | T5 | Transcriptional Terminator | Consists of B0010 (T1 from *E. coli* rrnB) and B0012  (transcription terminator for the *E. coli* RNA polymerase) |
| B0014 | T6 | Transcriptional Terminator | Consists of B0012(transcription terminator for the *E. coli* RNA polymerase) and B0011 |
| J18963 | T7 | Transcriptional Terminator | rpn terminator from DNA2.0 plasmid |
| B0025 | T8 | Transcriptional Terminator | Reverse sequence of B0015 |

Note: the data referred to *ACS Synthetic Biology.* 2013, 2, 9, 506–518 and *Nucleic Acids Research* 25, 1203–1210.

**Table S2.** The selected RBS sequences

| No. | Sequence (5’-3’) |
| --- | --- |
| AR1 | CCAGTTGTATGGAAATAGTAAAAAAAAGGGTAAGGAGGTATATAT |
| AR2 | GCACACCGGGAGGAAGAAGTTAAGGAGGTATTATT |
| AR3 | TTGGCGACGAATTAAGGAGATTTTTT |
| AR4 | TGACTCTAAATTGAGTCGAGGGGAATTAGGATACTAAGGAGGATTTAT |
| AR5 | TCTAACCTAGAATTTAAGAACTATAAGGAGGTATTTT |
| AR6 | TTAGACTCTACCAAGGAGATAGGAAAATAAGGAGGTATTAAT |
| AR7 | ACGTATTACACACGACACCAAGTCTTAGAAGGAGGTAATTAC |

**Table S3** Calculated energy for RBS sequence

| No | Translation  Rate | *∆G_total_*  (Kcal/mol) | *∆G_mRNA:rRNA_*  (Kcal/mol) | *∆G_spacing_*  (Kcal/mol) | *∆G_standby_*  (Kcal/mol) | *∆G_start_*  (Kcal/mol) | *∆G_mRNA_*  (Kcal/mol) |
| --- | --- | --- | --- | --- | --- | --- | --- |
| AR1 | 457293.09 | -13.14 | -14.75 | 0.29 | 0.00 | -2.76 | -4.72 |
| AR2 | 487953.90 | -13.29 | -13.25 | 0.29 | 0.00 | -2.76 | -3.34 |
| AR3 | 75515.22 | -9.14 | -8.86 | 0.00 | 0.20 | -2.76 | -3.32 |
| AR4 | 248405.71 | -11.79 | -17.4 | 0 | 0 | -2.76 | -9.15 |
| AR5 | 288450.48 | -12.12 | -13.19 | 0.00 | 0.00 | -2.76 | -4.74 |
| AR6 | 467489.42 | -13.19 | -15.02 | 0.29 | 0 | -2.76 | -4.99 |
| AR7 | 395068.78 | -12.82 | -12.61 | 0.29 | 0.00 | -2.76 | -2.77 |

****Table S4.** Kinetic parameters of representative GDH, FDH, ADH toward NAD+**

| Variant | Substrate | *K*_m_  (mM) | *k*_cat_  (s^-1^) | *k*_cat_/*K*_m_  (mM^-1^s^-1^) | Reference |
| --- | --- | --- | --- | --- | --- |
| *Ss*GDH | NAD^+^ | 1.2 | 48 | 40 | *AMB Expr* 2015, 5, 68 |
| *Bm*GDH | NAD^+^ | 0.37 | 260 | 702 | *AMB Expr* 2015, 5, 68 |
| *Pse*FDH^A198G+D221S^ | NAD^+^ | 0.54±0.04 | 5.00±0.20 | 9.26 | *J Biol Inorg Chem*, 2018, 23(8): 1243-1254. |
| *Cbo*FDH^D195S+Y196P^ | NAD^+^ | 0.19±0.03 | 3.14±0.13 | 16.02 | *ACS Catal*, 2020, 10(14): 7512-7525 |
| *Gst*ADH^WT^ | NAD^+^ | 0.76±0.003 | 8.28±0.006 | 10.86 | This study |
| *Gst*ADH^S284T^ | NAD^+^ | 0.69±0.27 | 9.24±0.24 | 13.39 | This study |
| *Gst*ADH^E107S^ | NAD^+^ | 0.36±0.23 | 6.35 ±0.12 | 17.75 | This study |
| *Gst*ADH^E107S+S284T^ | NAD^+^ | 0.39±0.31 | 8.89±0.24 | 22.81 | This study |

Table S5 All primers used for alanine scanning

| Primer | Nucleotide sequence (5’→3’) |
| --- | --- |
| T237A-F | TGTAgcgGCTGTTTCCAAACC |
| T237A-R | CAGCcgcTACAACCGCGGCGTGA |
| V260A-F | TCTGgcgGGTCTGCCGCCGGA |
| V260A-R | GACCcgcCAGAACACAAGCACC |
| G326A-F | GAAAgcgCAGATCAACGGCCG |
| G326A-R | TCTGcgcTTTCAGCATGCGGTCG |
| G101A-F | GTCCgcgCAGGAAACTCTGT |
| G101A-R | CCTGcgcGGACAGGCAATAATC |
| L99A-F | TTGCgcgTCCGGTCAGGAAAC |
| L99A-R | CGGAcgcGCAATAATCGCAA |
| G283A-F | CATCgcgTCCATCGTTGGTAC |
| G283A-R | TGGAcgcGATGATTTTAATAC |
| G73A-F | TCCAgcgGTCACCCATCTGAA |
| G73A-R | TGACcgcTGGACCCACTTCTTCA |
| T160A-F | GGTTgcgGGTGCCAAACCAGGT |
| T160A-R | CACCcgcAACCTTCAGCGCTTT |
| V239A-F | TGCTgcgTCCAAACCAGCAT |
| V239A-R | TGGAcgcAGCAGTTACAACCGC |
| I328A-F | TCAGgcgAACGGCCGTGTTGTC |
| I328A-R | CGTTcgcCTGACCTTTCAGCATGC |
| G175A-F | CGGTgcgCTGGGTCACGTT |
| G175A-R | CCAGcgcACCGATACCGTAGA |

Table S6 All primers used for site-saturation mutagenesis

| Primer | Nucleotide sequence(5’→3’) |
| --- | --- |
| C148-F | TTTTCNNKGCGGGTGTGACCAC |
| C148-R | CCGCMNNGAAAATCGGAGCAGCTTC |
| I285-F | TTCCNNKGTTGGTACTCGTAAG |
| I285-R | CAACMNNGGAACCGATGATTTTA |
| V286-F | CCATCNNKGGTACTCGTAAGG |
| V286-R | TACCMNNGATGGAACCGATGATT |
| S284-F | CGGTNNKATCGTTGGTACTCG |
| S284-R | CGATMNNACCGATGATTTTAATA |
| R331-F | CGGCNNKGTTGTCCTGACGCTG |
| R331-R | CAACMNNGCCGTTGATCTGACC |
| W87-F | CCCTNNKCTGTATAGCGCATG |
| T152-F | TGTGNNKACCTACAAAGCGCTGA |
| T152-R | AGGTMNNCACACCCGCMNNGAAAA |
| L262-F | TGGTNNKCCGCCGGAGGAAAT |
| L262-R | GCGGMNNACCAACCAGAACACAAG |
| H39-F | CTGTNNKACCGATCTGCATGC |
| H39-R | CGGTMNNACAGACACCACACGC |
| E107-F | GTGCNNKCACCAGAAGAATGCGG |
| E107-R | GGTGMNNGCACAGAGTTTCCTGA |
| T104-F | GGAANNKCTGTGCGAACACCA |
| T104-R | ACAGMNNTTCCTGACCGGACAG |


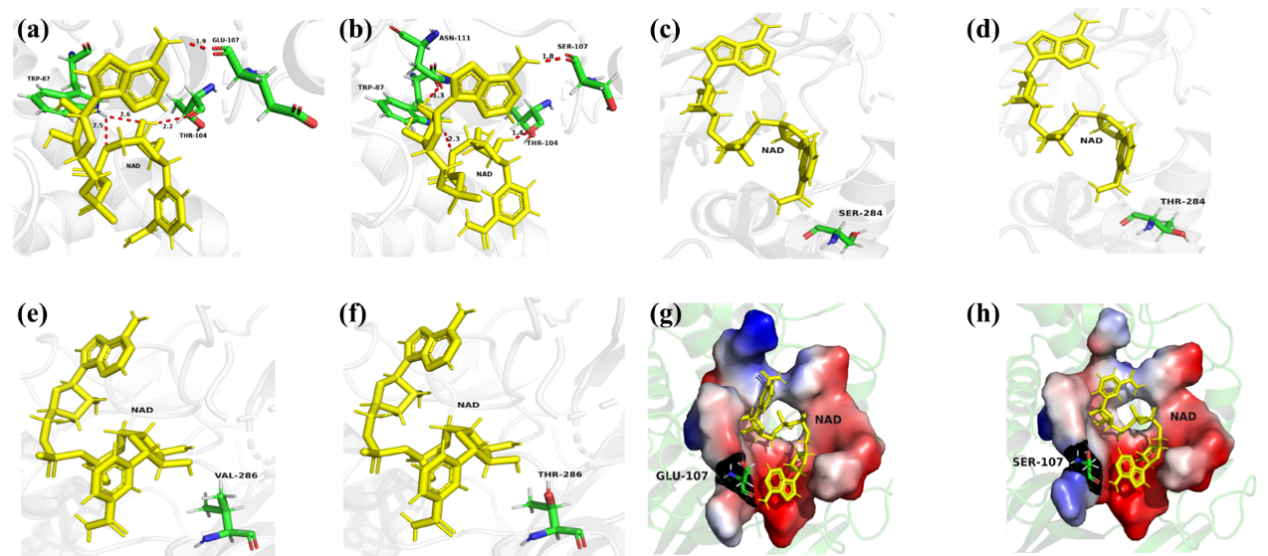


**Figure S1**. The locations of the mutant in *Gst*ADH


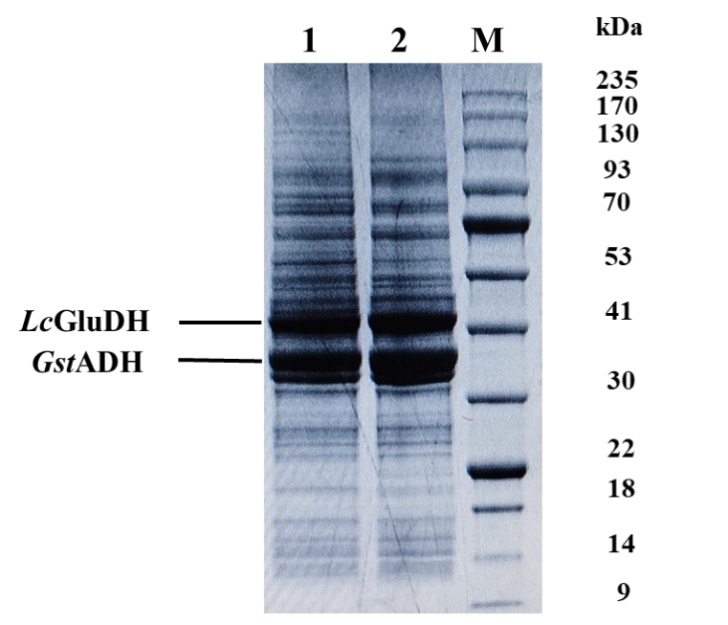


**Figure S2**. SDS-PAGE analysis of *Lc*GluDH and *Gst*ADH co-expressed in *E. coli* BL21(DE3). Lane 1: *Lc*GluDH and *Gst*ADH^WT^ co-expressed in *E. coli* BL21(DE3); Lane 2: *Lc*GluDH and *Gst*ADH^E107S/S284T^ co-expressed in *E. coli* BL21(DE3); Lane M: Marker


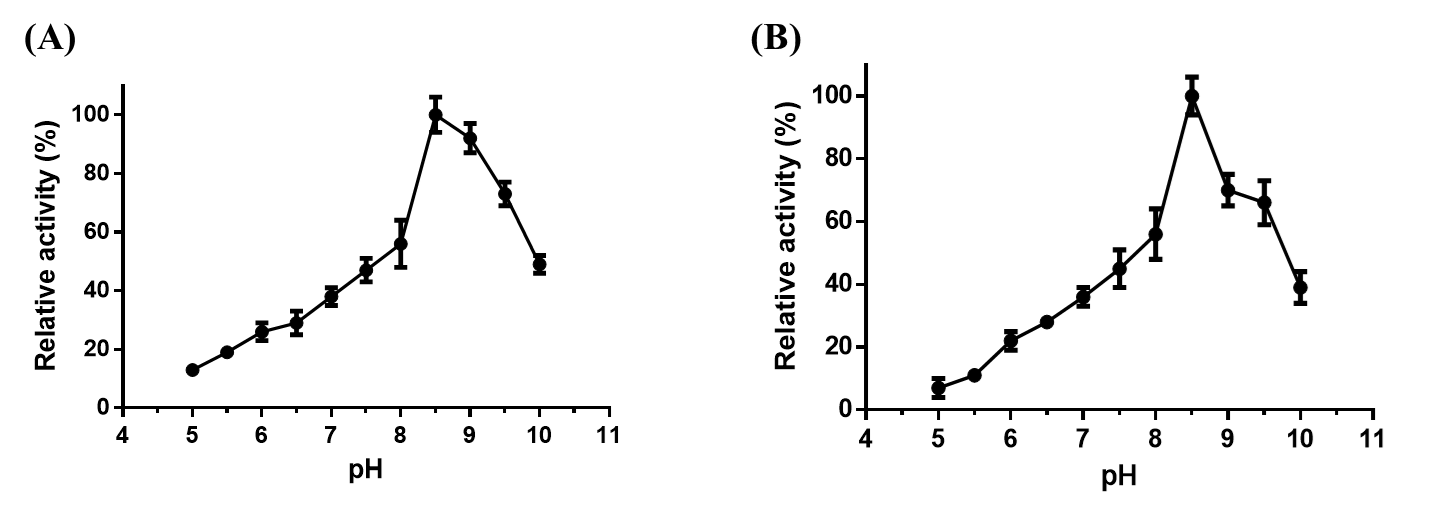


**Figure S3**. The pH effects on ADH-based NADH regeneration system. (A) The pH effects on *Gst*ADH^WT^-based NADH regeneration system; (B) The pH effects on *Gst*ADH^E107S/S284T^-based NADH regeneration system


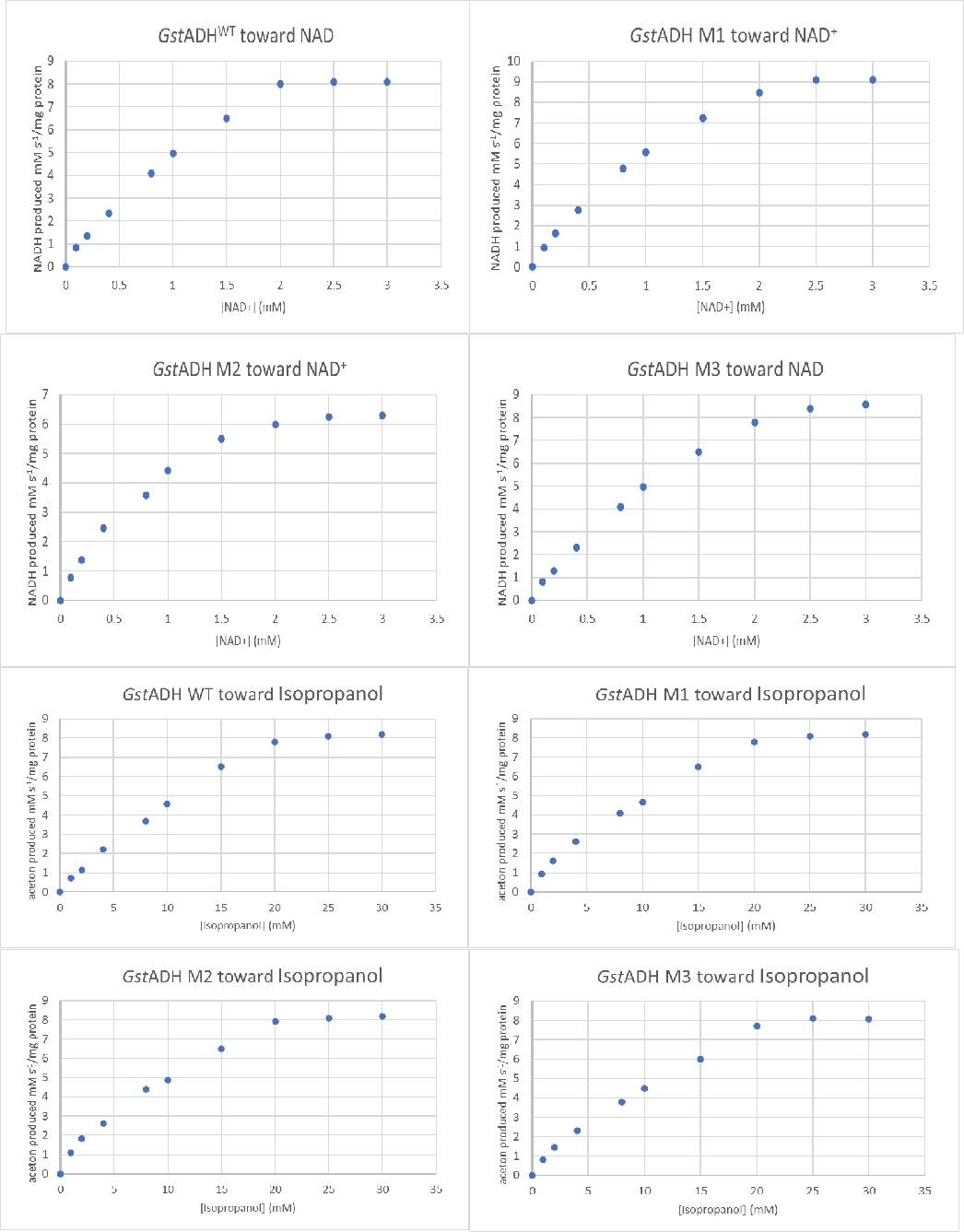


**Figure S4**. Kinetic curve of *Gst*ADH and its variants.


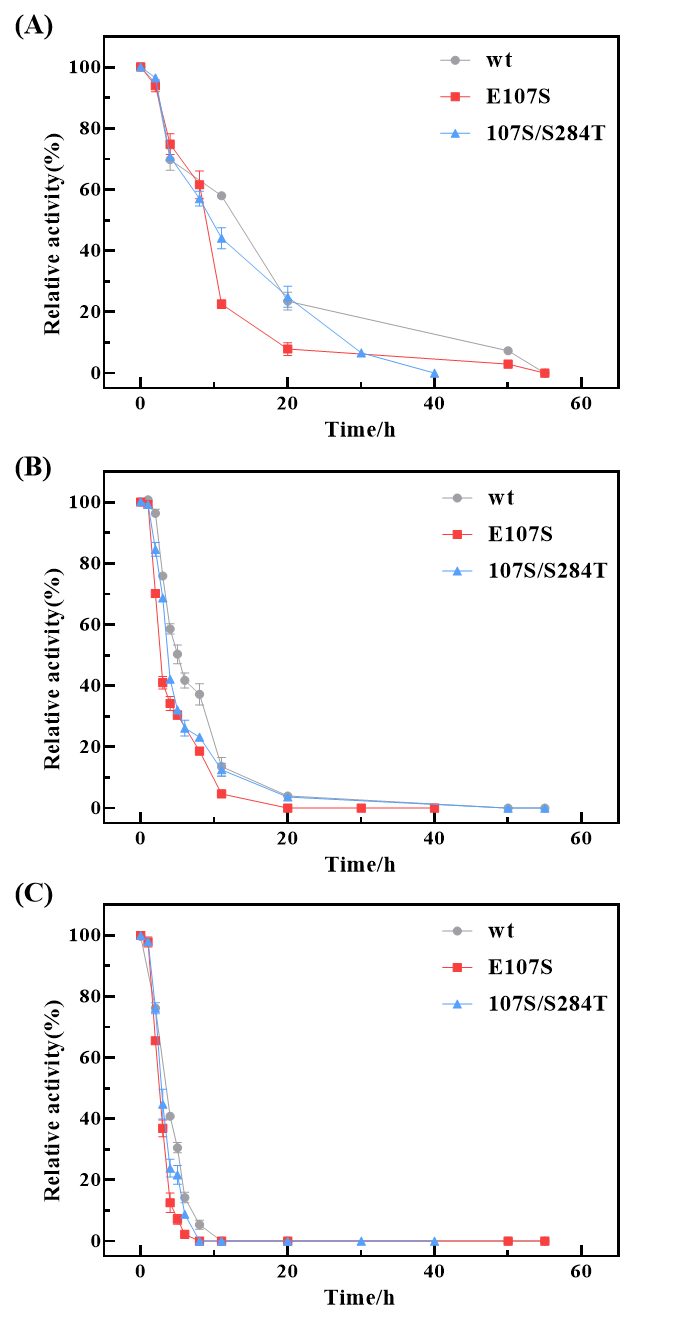


**Figure S5.** The thermal stability of *Gst*ADH and its variants. (A) *Gst*ADH^WT^, (B) *Gst*ADH^E107S^, and (C) *Gst*ADH^E107S/S284T^. The activity is tested in different temperatures and pH 8.5.


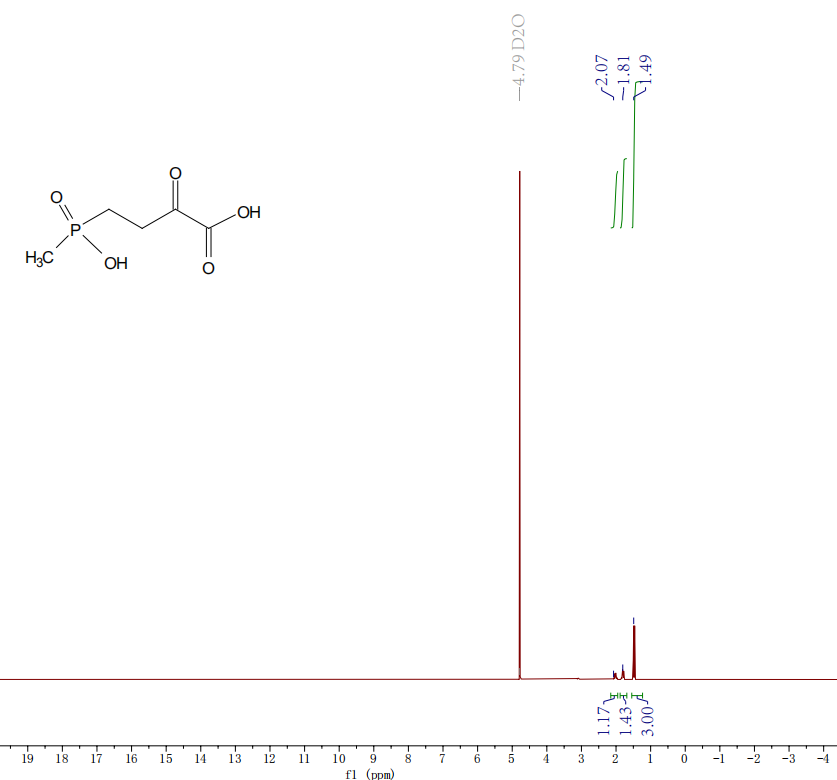


**A**


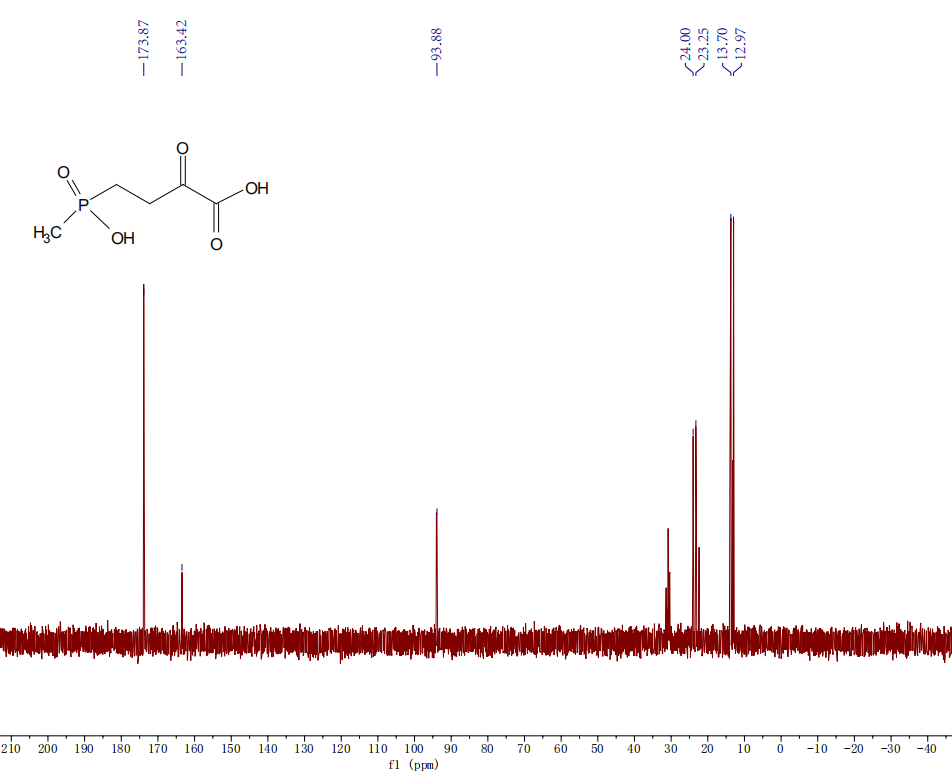


**B**

**Figure S6. NMR spectra of the synthesized substrate 2-oxo-4- {(hydroxy)(methyl)phosphinyl butyric acid (PPO).** (A) ^1^H NMR (500 MHz, Deuterium Oxide) δ 2.07 (s, 1H), 1.81 (s, 1H), 1.49 (s, 3H). (B) ^13^C NMR (126 MHz, D2O) δ 220.74, 173.87, 163.42, 93.88, 24.00, 23.25, 13.70, 12.97.


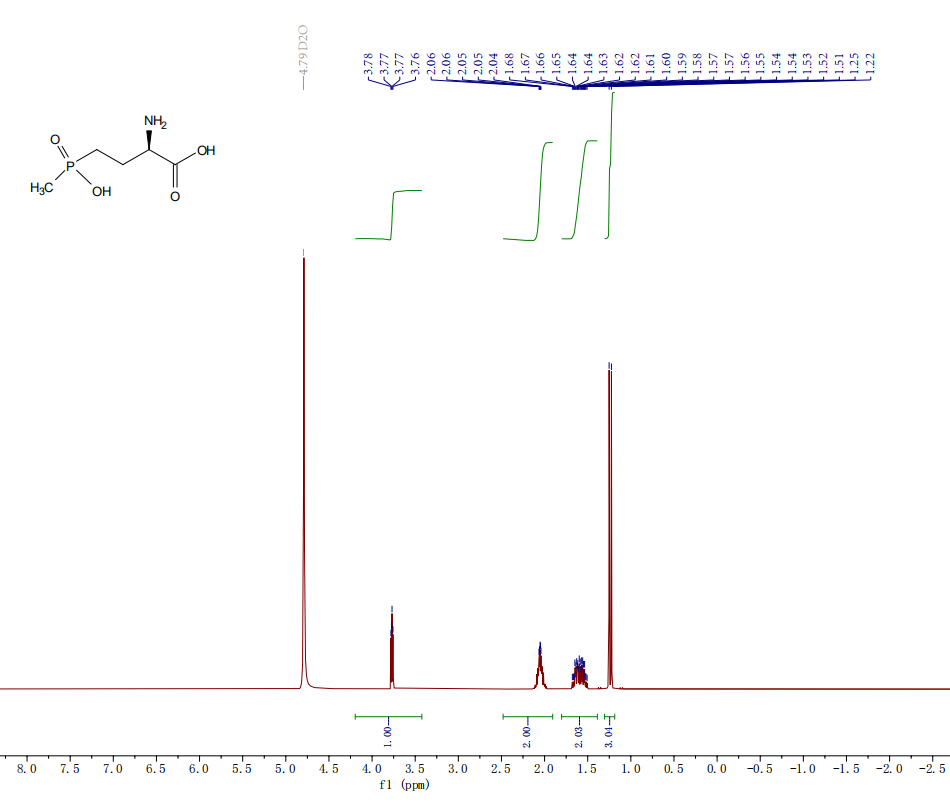


**A**

**
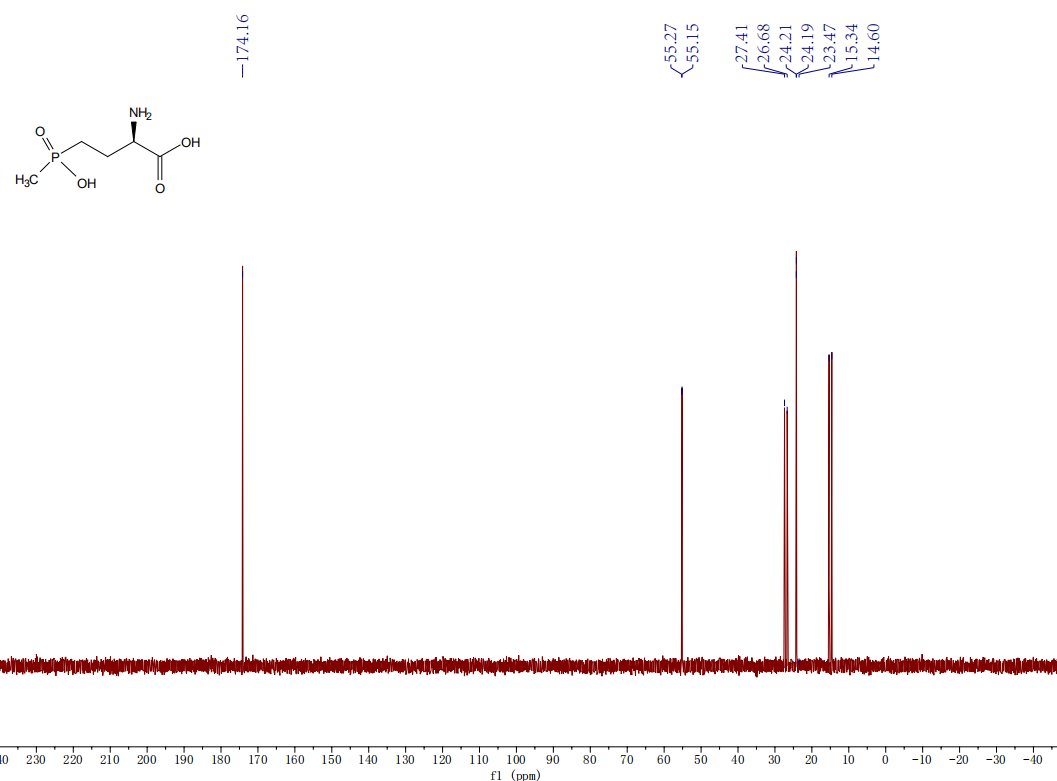
**

**B**

**Figure S7. NMR spectra of the purified L-phosphinothricin.** (A) ^1^H NMR (500 MHz, Deuterium Oxide) δ 2.12 – 2.02 (m, 1H), 1.86 – 1.77 (m, 1H), 1.49 (dd, *J* = 14.1, 6.7 Hz, 2H). (B)^13^C NMR (126 MHz, D_2_O) δ 174.16, 55.27, 55.15, 27.41, 26.68, 24.21, 24.19, 23.47, 15.34, 14.60.

>The sequence of wide-type ADH

MKAAVVEQFKEPLKIKEVEKPTISYGEVLVRIKACGVCHTDLHAAHGDWPVKPKLPLIPGHEGVGIVEEVGPGVTHLKVGDRVGIPWLYSACGHCDYCLSGQETLCEHQKNAGYSVDGGYAEYCRAAADYVVKIPDNLSFEEAAPIFCAGVTTYKALKVTGAKPGEWVAIYGIGGLGHVAVQYAKAMGLNVVAVDIGDEKLELAKELGADLVVNPLKEDAAKFMKEKVGGVHAAVVTAVSKPAFQSAYNSIRRGGACVLVGLPPEEMPIPIFDTVLNGIKIIGSIVGTRKDLQEALQFAAEGKVKTIIEVQPLEKINEVFDRMLKGQINGRVVLTLEDK

> *Gst*ADH^S284T^ (M1)

MKAAVVEQFKEPLKIKEVEKPTISYGEVLVRIKACGVCHTDLHAAHGDWPVKPKLPLIPGHEGVGIVEEVGPGVTHLKVGDRVGIPWLYSACGHCDYCLSGQETLCEHQKNAGYSVDGGYAEYCRAAADYVVKIPDNLSFEEAAPIFCAGVTTYKALKVTGAKPGEWVAIYGIGGLGHVAVQYAKAMGLNVVAVDIGDEKLELAKELGADLVVNPLKEDAAKFMKEKVGGVHAAVVTAVSKPAFQSAYNSIRRGGACVLVGLPPEEMPIPIFDTVLNGIKIIGTIVGTRKDLQEALQFAAEGKVKTIIEVQPLEKINEVFDRMLKGQINGRVVLTLEDK

>*Gst*ADH^E107S^ (M2)

MKAAVVEQFKEPLKIKEVEKPTISYGEVLVRIKACGVCHTDLHAAHGDWPVKPKLPLIPGHEGVGIVEEVGPGVTHLKVGDRVGIPWLYSACGHCDYCLSGQETLCSHQKNAGYSVDGGYAEYCRAAADYVVKIPDNLSFEEAAPIFCAGVTTYKALKVTGAKPGEWVAIYGIGGLGHVAVQYAKAMGLNVVAVDIGDEKLELAKELGADLVVNPLKEDAAKFMKEKVGGVHAAVVTAVSKPAFQSAYNSIRRGGACVLVGLPPEEMPIPIFDTVLNGIKIIGSIVGTRKDLQEALQFAAEGKVKTIIEVQPLEKINEVFDRMLKGQINGRVVLTLEDK

>*Gst*ADH^E107S+S284T^ (M3)

MKAAVVEQFKEPLKIKEVEKPTISYGEVLVRIKACGVCHTDLHAAHGDWPVKPKLPLIPGHEGVGIVEEVGPGVTHLKVGDRVGIPWLYSACGHCDYCLSGQETLCSHQKNAGYSVDGGYAEYCRAAADYVVKIPDNLSFEEAAPIFCAGVTTYKALKVTGAKPGEWVAIYGIGGLGHVAVQYAKAMGLNVVAVDIGDEKLELAKELGADLVVNPLKEDAAKFMKEKVGGVHAAVVTAVSKPAFQSAYNSIRRGGACVLVGLPPEEMPIPIFDTVLNGIKIIGTIVGTRKDLQEALQFAAEGKVKTIIEVQPLEKINEVFDRMLKGQINGRVVLTLEDK
